# Participatory disease surveillance for a mass gathering — a prospective cohort study on COVID-19, Germany 2021

**DOI:** 10.1186/s12889-022-14505-x

**Published:** 2022-11-14

**Authors:** Nils Hohmuth, Ifrah Khanyaree, Anna-Lena Lang, Ohad Duering, Stefan Konigorski, Vukašin Višković, Tobias Heising, Friedemann Egender, Cornelius Remschmidt, Rasmus Leistner

**Affiliations:** 1 Data4Life gGmbH, Charlottenstraße 13, 10969 Berlin, Germany; 2 Medizinische Klinik für Gastroenterologie-, Infektiologie-, und Rheumatologie, Charité University Medicine Berlin, Campus Benjamin Franklin, Berlin, Germany; 3 Digital Health Center, Hasso Plattner Institute for Digital Engineering, University of Potsdam, Potsdam, Germany; 4 Hasso Plattner Institute for Digital Health at Mount Sinai, Icahn School of Medicine at Mount Sinai, New York, USA; 5 Medizinisches Versorgungszentrum Bohmte, Bremer Str. 37, 49163 Bohmte, Germany; 6 Medizinische Klinik für Nephrologie und Intensivmedizin, Charité University Medicine Berlin, Campus Virchow, Berlin, Germany

**Keywords:** COVID-19, SARS-CoV-2, Coronavirus, Mass gathering, Epidemiology, Infectious diseases, Participatory disease surveillance, Syndromic surveillance, Mobile app, Web application

## Abstract

**Background:**

Mass gatherings (MGs) such as music festivals and sports events have been associated with a high risk of SARS-CoV-2 transmission. On-site research can foster knowledge of risk factors for infections and improve risk assessments and precautionary measures at future MGs. We tested a web-based participatory disease surveillance tool to detect COVID-19 infections at and after an outdoor MG by collecting self-reported COVID-19 symptoms and tests.

**Methods:**

We conducted a digital prospective observational cohort study among fully immunized attendees of a sports festival that took place from September 2 to 5, 2021 in Saxony-Anhalt, Germany. Participants used our study app to report demographic data, COVID-19 tests, symptoms, and their contact behavior. This self-reported data was used to define probable and confirmed COVID-19 cases for the full “study period” (08/12/2021 – 10/31/2021) and within the 14-day “surveillance period” during and after the MG, with the highest likelihood of an MG-related COVID-19 outbreak (09/04/2021 – 09/17/2021).

**Results:**

A total of 2,808 of 9,242 (30.4%) event attendees participated in the study. Within the study period, 776 individual symptoms and 5,255 COVID-19 tests were reported. During the 14-day surveillance period around and after the MG, seven probable and seven PCR-confirmed COVID-19 cases were detected. The confirmed cases translated to an estimated seven-day incidence of 125 per 100,000 participants (95% CI [67.7/100,000, 223/100,000]), which was comparable to the average age-matched incidence in Germany during this time. Overall, weekly numbers of COVID-19 cases were fluctuating over the study period, with another increase at the end of the study period.

**Conclusion:**

COVID-19 cases attributable to the mass gathering were comparable to the Germany-wide age-matched incidence, implicating that our active participatory disease surveillance tool was able to detect MG-related infections. Further studies are needed to evaluate and apply our participatory disease surveillance tool in other mass gathering settings.

**Supplementary Information:**

The online version contains supplementary material available at 10.1186/s12889-022-14505-x.

## Background

At mass gatherings (MGs), the risk of SARS-CoV-2 transmission can be substantially increased [1–7]. Therefore, early in the COVID-19 pandemic MGs were prohibited in most countries and later re-approved under strict precautionary measures [8]. Appropriate hygiene concepts became mandatory with the involvement of event organizers, epidemiologists, and the responsible local health authorities [9]. To improve the safety of MGs and the effectiveness of hygiene concepts and to reduce the risk of MG-related outbreaks, subsequent scientific evaluations are necessary [2–7, 10–12]. The duration of MGs, the location (e.g., indoor or outdoor), and the compliance with precautionary measures such as social distancing and mask use have been shown to influence MG-related COVID-19 outbreaks; however, most of MG outbreak research was conducted retrospectively by using routine surveillance data or interviews [10–13]. A review of prospective disease surveillance tools for MGs published in 2022 showed that most concepts rely on facility-based health care data to identify MG-related infections. Almost half of the studies used questionnaires carried out by healthcare staff during visits of sick event attendees [14]. The review indicates that current surveillance methods were not reliable for detecting potential MG-related outbreaks. Only a few MGs were accompanied by randomized controlled trials to assess COVID-19 infection rates and associated risk factors in more detail [7, 15, 16]. Such study designs can be costly and difficult to implement within the organizational structure of MGs. Furthermore, they may alter the experience of these cultural events.

In search of scalable and easy to implement solutions to identify infection trends, more recent concepts rely on “participatory disease surveillance” with self-reported data from event attendees [17–21]. This crowdsourcing approach could help to simplify the early detection of MG-related infections and further improve knowledge on infection risk factors at MGs [8, 22]. In the above-mentioned review only one of 19 strategies (5.2%) relied on this form of citizen science [14, 20]. Prior to the COVID-19 pandemic, participatory disease surveillance has been conducted at the Hajj in 2014, the FIFA World Cup in 2014, and the Olympics in 2016 using desktop- or mobile apps to prospectively collect symptom data from event attendees. Theses existing participatory disease surveillance tools for MGs utilized self-reported symptom data but not testing data to identify infections [17–20]. We developed a web- and mobile application (herein called “study app”) that facilitates the prospective collection of COVID-19 related data at high scale while ensuring data privacy and usability. The goal of this study was to explore whether self-reported COVID-19 symptoms and testing data can be used to identify MG-related COVID-19 infections.

## Methods

### Study design

**Fig. 1:**
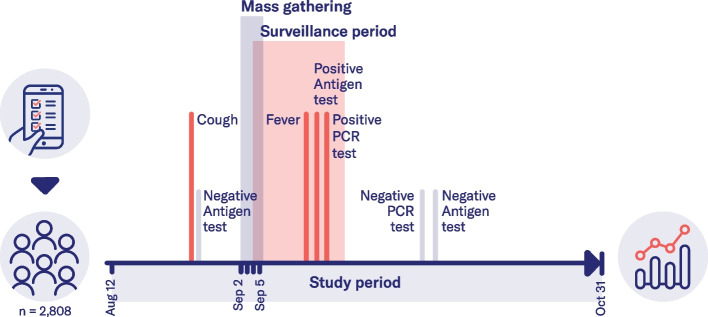
Overview of the mass gathering study concept**.** The figure shows the study concept and timeline. We explored the feasibility of a digital study app as a COVID-19 surveillance tool at a mass gathering (MG). 2,808 out of 9,242 event attendees enrolled in our study. Participants used our study app to report demographic information, contact behavior, COVID-19 symptoms, and test results during the study period. As visualized on the timeline, the study period ranged from 08/12 to 10/31/2021. The MG took place from 09/02 to 09/05/2021. We defined the surveillance period as the period of highest likelihood of MG-related COVID-19 cases (09/04/2021 – 09/17/2021). Examples of data reports from one participant are visualized on the timeline. Our analysis focused on assessing the feasibility of the study app as a surveillance tool by reporting the number of possibly MG-related SARS-CoV-2 infections within the surveillance period

**Fig. 2:**
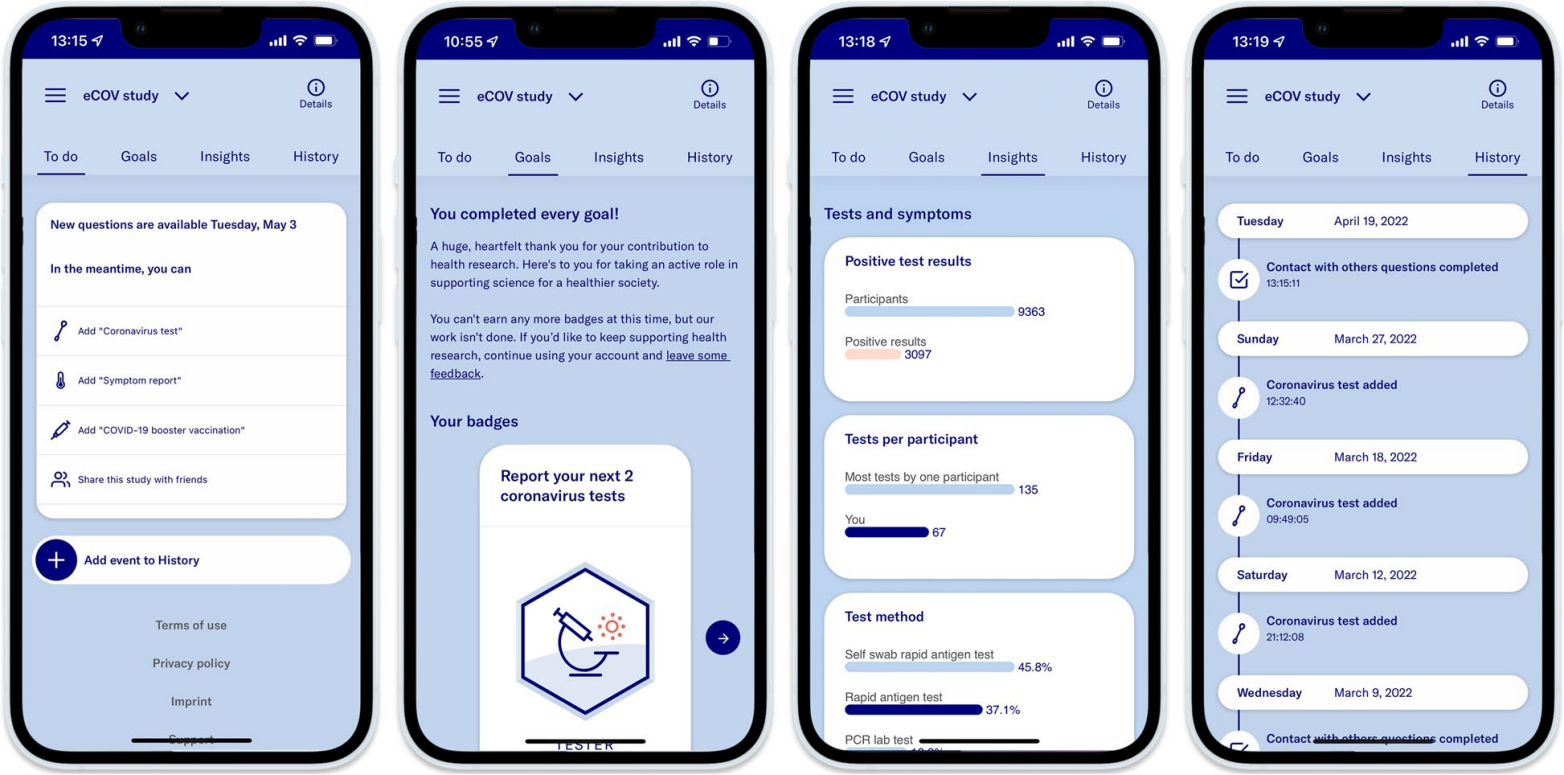
eCOV study app user interface. In-App screenshots of the eCOV study user interface

We conducted a digital prospective cohort study among attendees of an MG that took place from September 2 to 5, 2021 (see Fig. 1 and section “Mass gathering setting” for more details). Prior to the MG, the event organizers promoted the study through emails and their event website as well as social media channels. Enrolment was open to all event attendees during the predefined study period (08/12/2021 – 10/31/2021). Participants were asked to fill out different questionnaires that were provided in the study app (Fig. 2). Upon enrolment, participants were asked to fill out a demographic- and vaccination questionnaire. On-demand test and symptom questionnaires enabled participants to report COVID-19 tests and symptoms at any time. A monthly questionnaire asked about contact behavior and exposure to COVID-19 cases and reminded study participants to report their tests and symptoms. Email notifications reminded the participants about this questionnaire and the ongoing of the study. During the MG, study posters on-site reminded the participants to report their data to the study app. Correction or deletion of answers in the app was possible within 48 h. Five study-related challenges were used as a gamification feature to motivate study participants to report all COVID-19 test results and symptoms in the app. When completing these voluntary challenges such as reporting a certain number of tests or completing monthly questionnaires, participants were rewarded with digital badges and shown information on the scientific value of their data contribution (Fig. 2). Monetary incentives were not provided.

### Mass gathering setting

The MG surveilled in this publication is an annual outdoor sports festival, hosting mainly medical students and doctors from different European countries. In 2021, during the COVID-19 pandemic, this MG was approved under strict precautionary measures and took place from September 2 to 5 at an outdoor area of 45 hectares in the federal state of Saxony-Anhalt, Germany, gathering 9,242 event attendees and around 500 staff members. Only individuals with proof of full immunity according to the specifications of the national public health institute (Robert Koch-Institut, RKI) were granted access [23]. This included individuals that either 1) had recovered from a past COVID-19 infection that occurred > 4 weeks and < 6 months prior to the MG with or without further vaccination AND/OR 2) had recovered from a past COVID-19 infection that occurred > 6 months prior to the MG with vaccination AND/OR 3) were fully vaccinated with one or more vaccines approved by the European Medicines Agency (EMA) or equivalents of these vaccines used in non-EU countries > 2 weeks before the MG. Further, upon entry individuals had to provide proof of a negative PCR test not older than 48 h or a negative antigen test not older than 24 h from an official COVID-19 test center. Attendees were only allowed to travel to the MG by car or public transport and shared tour buses were not allowed as part of the hygiene concept. Attendees camped on-site and mask use was not mandatory. During the MG, trained staff conducted nasal swabs amongst a random sample of event attendees and staff members, using the “Novel Coronavirus 2019-nCoV Antigen Test (Colloidal gold)” from Beijing Hotgen Biotech Co., Ltd. (AT120/20 AT1236/21). In case of a positive COVID-19 test, dedicated health care facilities were provided on-site for possibly infected individuals and their risk contacts.

### Case definition: Probable and confirmed COVID-19 cases

**Fig. 3:**
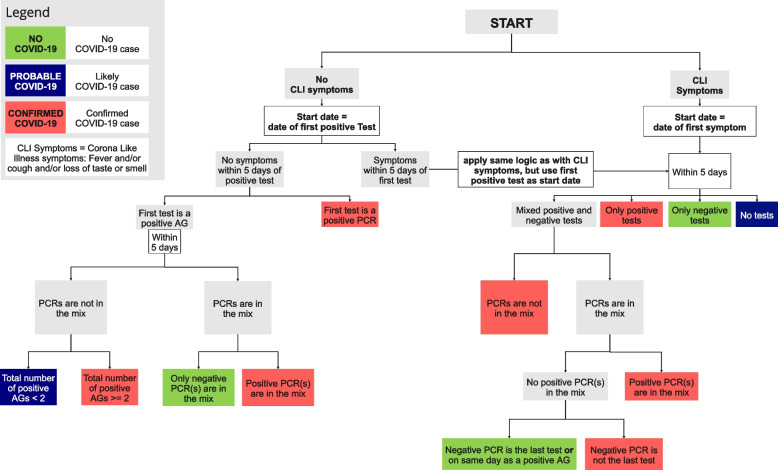
Flowchart of case definitions. Visualization of decision tree for case definitions used in this study (no COVID-19, probable COVID-19, and confirmed COVID-19)

We used self-reported COVID-19 symptoms in combination with test results to assign the study participants to one of the three case definitions: “no COVID-19”, “probable COVID-19” and “confirmed COVID-19”. The case definitions are based on definitions from the European Centre for Disease Prevention and Control (ECDC) [24, 25]. COVID-19 symptoms, hereafter called Corona-like illness (CLI) symptoms, were defined as: cough AND/OR fever AND/OR loss of taste or smell. COVID-19 tests that were considered for the case definitions included antigen and PCR tests, whereas reported blood tests or tests of unknown method were ignored. A detailed flow-chart in Fig. 3 visualizes the case definition including the time restrictions it incorporated. We assumed the COVID-19 delta variant (B.1.617.2) to be the predominant variant during the study period and incorporated knowledge of the specific incubation period and infectiousness in our case definitions [25–28]. The definition of a “confirmed COVID-19” case applied to individuals that 1) reported a positive PCR test independent of symptoms OR 2) reported at least two positive antigen tests within 5 days OR 3) reported CLI symptoms AND at least one positive antigen test within 5 days of symptom onset. For participants with positive antigen tests only, we allowed a period of 5 days to report a negative PCR test revoking the suspicion of a positive COVID-19 case, defining the participant as “no COVID-19”. The definition of “no COVID-19” also applied to individuals who did not report symptoms AND/OR did not report any positive tests. The definition of a “probable COVID-19” case included individuals that 1) did not report symptoms but reported one positive antigen test AND no further tests thereafter OR 2) reported CLI symptoms but no tests. Probable and confirmed cases were declared as related to the MG if they were reported within the 14-day surveillance period of two days after the beginning of the MG to 12 days after the MG. This broad surveillance period was defined to account for delays in testing availability and outliers with long incubation periods [27, 29, 30]. Following definitions from the German public health institute RKI, a person declared as a confirmed or probable COVID-19 case stayed in this category for ten days after the first reported positive test or symptom onset. Reinfection was only possible after 60 days [31].

### Statistical analysis

We first calculated descriptive statistics. Continuous variables are presented as mean with standard deviation (SD), while categorical variables are presented as numbers (n) and percentages (%). Our descriptive analysis was carried out as an intention-to-treat approach, including all participants that signed up for the study and filled out the initial demographic questionnaire [32]. Due to the small number of COVID-19 cases in the surveillance period, we did not apply statistical methods to compare individual risk factors between confirmed COVID-19 cases and healthy individuals. To calculate the seven-day incidence, we filtered for confirmed cases that were PCR positive, aligning with the case definition of the RKI [33]. We estimated 95% Confidence Intervals (CI) using the method of Agresti and Coull for our estimated seven-day incidences. We descriptively compared the incidence of MG-related COVID-19 cases with the Germany-wide incidence provided by the RKI for calendar weeks 36 and 37 [33, 34]. We hereby calculated the age-matched Germany-wide incidence according to the proportions of ages present in our study sample. As only a small proportion of participants joined the MG from other countries (5.9%, see Table 1), we only used data on the Germany-wide age-matched incidence. Analyses were performed on our internal Data4Life Analytics Platform version 22.1 that hosted a Jupyter notebook configured with Python version 3.9.7 using pandas version 1.3.3 and numpy version 1.19.5. Plots were built using ggplot2 with R version 4.1.3.

**Table 1:** Demographics of study participants. If not stated differently, all numbers displayed are n (%). Percentages are calculated from *n* responses to each question. Age and BMI are displayed as means with standard deviation (SD). *COVID-19 immunity (vaccination and/or prior infection) as controlled at MG entrance 09/02/2021. **Displaying answers of participants that reported their full vaccine status only

Demographics	All responses
**Age in years, *n* = 2808**	
Mean (SD)	23.8 (± 2.7)
**Gender, *n* = 2798**	
Female	1747 (62.4%)
Male	1049 (37.5%)
Diverse	2 (0.1%)
**BMI in kg/m**^**2**^**, *n* = 2678**	
Mean (SD)	22.4 (± 3)
**Any chronic disease, *n* = 2635**	
Allergies	741 (28.1%)
Others	187 (7.1%)
**Immune deficiency, *n* = 2782**	
Immune deficiency	35 (1.3%)
**Smoking, *n* = 2784**	
Active smoker	180 (6.5%)
**Healthcare profession, *n* = 2735**	
Medical student	1231 (45%)
Hospital	702 (25.7%)
Doctors’ office	112 (4.1%)
Nursing facility or retirement home	12 (0.4%)
Other medical field	302 (11%)
Not health care sector	376 (13.7%)
**Residency, *n* = 2808**	
Germany	2633 (94.1%)
Abroad	165 (5.9%)
COVID-19 immunity*, *n* = 2808	
Full immunity	2808 (100%)
Type of COVID-19 vaccine**, *n* = 2062	
2 × BNT162b2 (BioNTech/Pfizer)	1224 (59.4%)
2 × mRNA-1273 (Moderna)	234 (11.3%)
2 × AZD1222 (ChAdOx1, AstraZeneca)	97 (4.7%)
1 × JNJ-78436735 (Ad26.COV2.S, Johnson&Johnson)	27 (1.3%)
2 × Any combination	480 (23.3%)

### Data source

The data presented in this publication was collected using the study app infrastructure of a prospective cohort study which assessed the real-world effectiveness of COVID-19 vaccines (eCOV study, registered at Deutsches Register für Klinische Studien, ID: DRKS00025169). The study and the study app were developed by the not-for-profit organization Data4Life (Berlin, Germany). The study analyzing the MG was open for enrollment and reporting of data during the whole study period from 08/12/2021 to 10/31/2021. All data points reported after 10/31/2021 were not part of the MG study dataset, but participants could continue to report data to the eCOV study until 08/01/2022.

### Data protection and ethical considerations

The study was approved by the ethics committee of the Berlin Chamber of Physicians (Eth-11/22). All procedures were carried out in accordance with relevant guidelines and regulations. Registration was open for participants aged 18 years and older. To voluntarily enroll in the study, participants created a Data4Life account agreeing to the terms and conditions of the study app. Email and password were required to log in to the study app. The study app could be accessed via web browser on both desktop and mobile devices (Fig. 2). To join the eCOV study they had to give informed digital consent for the use of their study data in COVID-19 research. Participants could revoke their consent at any time. All research data was end-to-end encrypted, pseudonymized and forwarded to the Data4Life Analytics Platform, where it was accessed and analyzed by authorized researchers. More detailed information about the used protocol can be found in the Data4Life Crypto Bluebook [35]. The processed study data is stored exclusively within Germany in certified Data4Life data centers. Data4Life is certified by the German Federal Office for Information Security (BSI) according to ISO 27001 based on IT-Grundschutz.

## Results

A total of 2,808 out of 9,242 event attendees (30.4%) enrolled in our study. Study participants were on average 23.8 years old (± 2.7, range 19–56 years), and the majority were female (62.4%, see Table 1). 86.2% of the participants had a medical background with 41.2% working in the health care sector and 45% medical students. Most participants were from Germany (94.1%) with only a few participants from other European countries. 47% of participants (*n* = 2,772) reported to be living in a household with three or more people. Most respondents of the monthly questionnaire (79% out of 2,759 answers) reported close contact (< 1.5 m distance for > 15 min) with more than six people outside of their personal living environment in the week of the inquiry. Concerning health status, 28.1% of participants stated to have allergies, and 1.3% of individuals reported having an immune deficiency. 95 out of 2,808 individuals (3,4%) reported a COVID-19 infection before the study period. 6.5% reported being active smokers. Other than that, individuals were believed to be healthy with a mean BMI of 22.4 (SD ± 3).

**Fig. 4:**
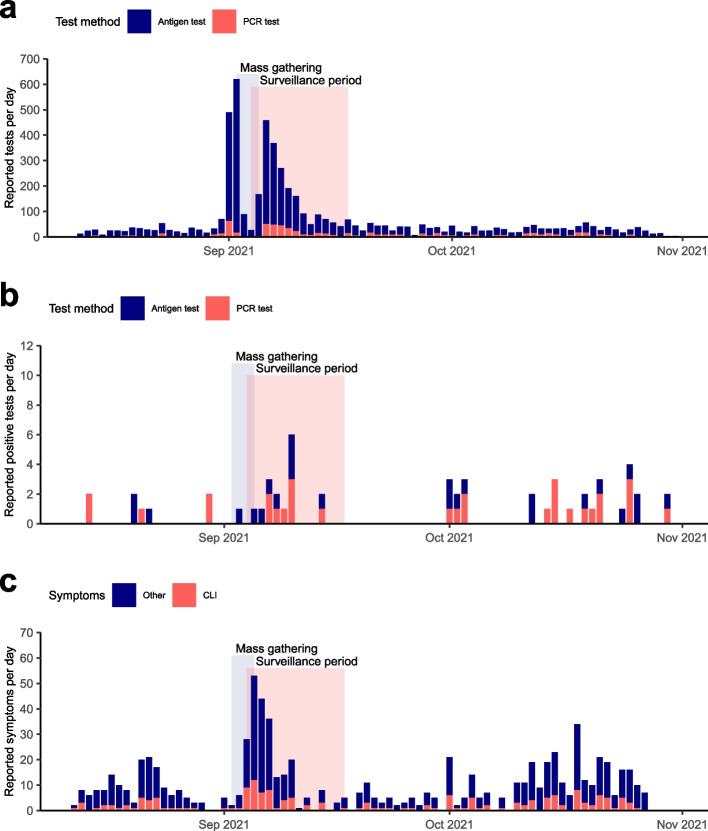
Reported tests and symptoms over the study period. Histogram plots display on the y-axis the number of **A** all reported COVID-19 tests by test method, **B** reported positive COVID-19 tests by test method, **C** reported symptoms split by corona-like-illness symptoms (CLI) and all other symptoms over the study period (08/12/2021 – 10/31/2021) on the x-axis. The MG period is highlighted in blue, spanning from 09/02/2022 to 09/05/2022. The highlighted surveillance period (09/04/2021 – 09/17/2021) reflects the period of high risk for occurrence of MG-related COVID-19 cases

During the study period, 209 participants reported a total of 776 symptoms and 1,787 participants reported a total of 5,255 COVID-19 tests. This translated to an average reporting of 0.3 symptoms and 1.9 tests per enrolled participant. All participants filled out the demographics questionnaire and 59% of participants filled out at least one of the maximum three monthly questionnaires asking about contact behavior and COVID-19 exposure. After the MG, we saw a decrease in reported tests and symptoms (Fig. 4). Participants that completed all three of the monthly questionnaires (n = 885) reported on average 1.3 symptoms, whereas participants that never reported any of the monthly questionnaires (n = 1,152) also did not report any symptoms. In addition, individuals that reported three of the monthly questionnaires on average reported 3.7 tests more than participants that never reported any of the monthly questionnaires (531.8% increase), indicating an association between the completion of the monthly questionnaire and the overall activity of participants in the study. The in-app feedback of 41 eCOV study participants during the study period (September and October 2021) resulted in a rating of 4.2 out of 5 stars.

**Fig. 5:**
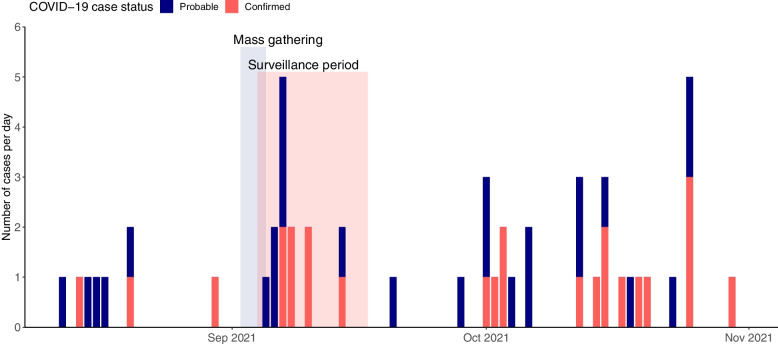
Epidemiological curve of probable and confirmed COVID-19 cases**.** Histogram plot displaying the number of confirmed and probable COVID-19 cases on the y-axis (based on our case definitions, see Methods) over the study period (08/12/2021 – 10/31/2021) on the x-axis. Color-coding reflects COVID-19 status (blue = probable, red = confirmed). The period of the MG is highlighted in blue, spanning from 09/02/2022 to 09/05/2022. The highlighted surveillance period (09/04/2021 – 09/17/2021) reflects the period of high risk for occurrence of MG-related COVID-19 cases

We detected a total of 24 PCR-confirmed COVID-19 cases and 26 probable COVID-19 cases during the entire study period. During the 14-day surveillance period (09/04/2021 – 09/17/2021) with COVID-19 cases attributable to the MG, we registered seven confirmed (PCR positive) and seven probable cases (Fig. 5). The seven confirmed COVID-19 cases per 2,808 participants translate to an estimated seven-day incidence of ~ 125 per 100,000 participants (95% CI [67.7/100,000, 223/100,000]), comparable to the average age-adjusted incidence in Germany during this time (calendar weeks 36 and 37: 118.3/100,000). Within the surveillance period, six probable and six confirmed COVID-19 cases were already registered in the first week, translating to an estimated seven-day incidence of ~ 213 confirmed cases per 100,000 participants (95%CI [135.8/100,000, 332.9/100,000]). In comparison, the age-adjusted incidence in Germany during this time was 131.9/100,000. During the second week of the surveillance period, one more probable and one more confirmed case was detected, translating to an estimated seven-day incidence of ~ 36 confirmed cases per 100,000 participants (95% CI [8/100,000, 104.8/100,000]). The test positive rate in our study cohort over the study period was below the Germany-wide test positive rate (Supplementary Fig. 1) [36]. Overall, the number of weekly COVID-19 cases detected in our app varied over the study period, with some calendar weeks before and after the MG (calendar weeks 34, 35, 38, 40) without confirmed cases and weeks where the seven-day incidence was comparable to the Germany-wide age-matched incidence (calendar weeks 36, 39, 41, 43).

Individuals classified as confirmed COVID-19 cases during the surveillance period were primarily female (85.7%, see Table 2) and on average 23.9 years old (SD ± 2.7). Four out of seven reported living outside of Germany. All confirmed cases in the surveillance period were fully vaccinated non-smokers and did not report any immune deficiencies. None of the confirmed cases were infected with COVID-19 before the study period and none of them reported any symptoms during the infection.

**Table 2:** Demographics by COVID-19 infection status. If not stated differently, all numbers are displayed as n (%). Percentages are calculated from n individuals in each group. CLI = Corona Like Illness defined as having fever AND/OR cough AND/OR loss of taste or smell

	**Total *n* = 2808**	**No COVID-19 *n* = 2794**	**Probable COVID-19 *n* = 7**	**Confirmed COVID-19 *n* = 7**
**Demographics**				
Age in years				
Mean (SD)	23.8 (± 2.7)	23.8 (± 2.7)	24.9 (± 4.2)	23.9 (± 2.7)
Gender				
Female	1747 (62.4%)	1738 (62.2%)	3 (42.9%)	6 (85.7%)
Male	1049 (37.5%)	1044 (37.4%)	4 (57.1%)	1 (14.3%)
Diverse	2 (0.1%)	2 (0.1%)	-	-
**Risk Profile**				
BMI in kg/m^2^				
Mean (SD)	22.4 (± 3)	22.4 (± 3)	22.1 (± 2.6)	21.9 (± 2.5)
Any chronic disease				
Allergies	741 (26.4%)	737 (26.4%)	2 (28.6%)	2 (28.6%)
Others	187 (6.6%)	185 (6.6%)	1 (14.3%)	1 (14.3%)
Immune deficiency				
	35 (1.2%)	35 (1.3%)	-	-
Active smoker				
	180 (6.4%)	178 (6.4%)	-	-
**Symptoms**				
Amount of symptoms				
CLI	53	48	5	-
Other	184	172	12	-

## Discussion

We showed that the self-reported symptoms and COVID-19 tests in our study app can be used to identify COVID-19 cases associated with an MG. As enrolment and data entry were self-administered by the study participants, data collection did not lead to additional efforts for the event organizers or the research team. Although participation in the study was voluntary and no incentives were given, almost a third of the event attendees (30.4%) enrolled in the study. This represents a broad fraction of event attendees compared to previously examined participatory surveillance tools for MGs [17–20]. Furthermore, other tools only collected self-reported symptom data [17–20]. The testing data collected in this study facilitated the identification of probable and confirmed COVID-19 cases through antigen tests as well as PCR tests as the gold standard for diagnosis of COVID-19 [37].

There are several factors that can help to validate the surveillance function of our tool. While the number of reported tests and symptoms varies over the study period, we found the highest number of tests and symptoms per day reported directly before the MG, reflecting the mandatory testing of event attendees before the MG. The number of COVID-19 cases detected during the surveillance period was comparable to the Germany-wide age-adjusted incidence during this time, though the estimated incidence within the first week after the MG was higher than the Germany-wide incidence. Therefore, the possibility of a minor increase of MG-associated infections exists. For the overall study period, reports of confirmed cases vary with some calendar weeks matching the Germany-wide incidence (calendar weeks 39, 41, 43) and some weeks without cases (calendar weeks 34, 35, 38, 40). This variance by weeks might indicate underreporting or reflect the small sample size of our cohort.

Apart from the use of disease surveillance for MGs in research, national health authorities make use of different tools to identify MG-associated infection clusters and warn a potential contact person. For routine analog contact tracing, public health authorities use retrospective data from notifiable diseases databases to reach out to infected individuals and interview them about the potential origin of their infection. If multiple infected individuals report the same MG as the potential origin of infection, clusters can be identified and further investigated. This process takes time and may require the coordination of a multitude of local health authorities on a national level. For international MGs, such as the one surveilled in our study, communication with foreign health authorities may be required. According to personal information provided to us by the event organizers, four MG-associated PCR-confirmed COVID-19 cases of 9,424 event attendees were reported to the responsible local health authorities, with two reported cases from one foreign health authority. We were not able to get official proof of this number. Through our tool we detected seven confirmed cases out of 2,808 event attendees. Four of the seven infected individuals reported being residents from two foreign countries. The reason why we found more COVID-19 cases remains unclear. It is possible that not all cases were officially reported to the responsible German health authorities (underreporting) and that our active surveillance tool was able to detect more cases than the passive routine surveillance by public health authorities. This could especially be true for the reported cases from participants that reside outside of Germany.

For the first time during the COVID-19 pandemic, digital tools such as Bluetooth exposure notifications apps (e.g., Corona-Warn-App) or digital guest list apps (e.g., Luca App) were broadly adopted to support traditional contact tracing efforts [38]. Both technologies aim to identify and notify app users that were in proximity to other app users that tested positive for COVID-19 [39]. In the context of MGs these tools show limitations. Due to privacy concerns, Bluetooth exposure notification apps do not collect information on the geolocation and thus COVID-19 cases cannot be attributed to MGs [40]. Digital guest lists aim to notify all users that checked in to the same venue, if any of the participating users reported to be infected. Such digital guest lists can be useful for smaller venues, but as positive cases lead to notification of all app users that checked in at the venue, they are impractical for MGs that go on for several days and host a large number of people. Finally, data from both tools is not accessible for research.

Our data suggests that participatory disease surveillance through our study app was a feasible method for COVID-19 surveillance at the MG. In future settings, our surveillance tool could include real-time dashboards designed for event organizers and health authorities to timely inform them about MG-associated infection trends. Our tool could further be improved by automated collection of testing data from official test sites. Using our surveillance tool in different MG settings could help to better understand the role of event characteristics in COVID-19 transmissions and thereby strengthen existing risk assessment tools and facilitate the safe planning of future MGs [4, 13, 41, 42].

## Limitations

Our study has several limitations. To put our findings into context, we compared our MG-related incidence of COVID-19 cases with the age-matched 7-day incidence from the national surveillance system. Different to the nation-wide data, our cohort was fully immunized and most likely had a high degree of health literacy as 86.2% of participants had a medical background. By its nature, all data was self-reported and 33.9% of the study participants did not report COVID-19 tests, which might have led to an underestimation of COVID-19 cases during the study period. Still, four of the seven confirmed cases (57.1%) and two of the seven probable cases (14.3%) detected during the surveillance period enrolled after the MG. This indicates an awareness about the study and its purpose amongst the event attendees. We did not enroll all event attendees to our study (30.4%), which might have introduced a selection bias. COVID-19 cases outside of our sample might have been missed or we may have caught the only infection clusters of the MG, thereby overestimating the total number of cases associated with the MG. In the context of our intention-to-treat analysis, not reporting tests or symptoms was equally weighted as reporting negative tests OR having no symptoms. As COVID-19 infections in our sample were rather infrequent, zero inflation must be considered when interpreting our results [43]. The fact that test positive rate in our study cohort was below the Germany-wide test positive rate (Supplementary Fig. 1) could be due to underreporting or influenced by the young age of our cohort, as we were not able to compare to an age-stratified dataset for Germany [36].

Amongst the seven confirmed COVID-19 cases within the surveillance period, none reported symptoms. It remains uncertain if these cases, who were infected but fully vaccinated, did not report their symptoms or had an asymptomatic infection. We assume that the MGs testing strategy and the event attendees awareness about the study led to increased testing as well as reporting of tests and symptoms to our app. Seroepidemiological COVID-19 studies from Germany identified an underreporting factor of at least two [44]. This underreporting might have been decreased amongst our MG study cohort, implying an even lower test positive rate.

## Conclusion

In this digital prospective cohort study, we used a participatory surveillance approach to collect COVID-19 related outbreak data for an MG. Our surveillance tool detected MG-related COVID-19 cases that were comparable to the age-matched Germany-wide incidence during that time, indicating an appropriate surveillance function of our tool. Our tool was used by almost a third of the event attendees and easy to implement within the organizational structure of the MG. In future settings, our participatory surveillance tool could be used in different MG scenarios to detect and analyze MG-related infection trends in real-time. This could help to timely inform health authorities and event organizers about possible outbreaks and adjust precautionary measures.

## Supplementary Information

**Additional file 1:**

## Data Availability

To ensure data protection and privacy of our research participants, the data supporting the findings of this study is not publicly available. Data can be made available from the corresponding author NH upon reasonable request.
