# Supplementary figures and images for: Participatory disease surveillance for a mass gathering — a prospective cohort study on COVID-19, Germany 2021
Source: BMC Public Health. 2022 Nov 14;22:2074. doi: 10.1186/s12889-022-14505-x (PMC9660174; doi:10.1186/s12889-022-14505-x)

— Test positivity rate mass gathering — Test positivity rate Germany

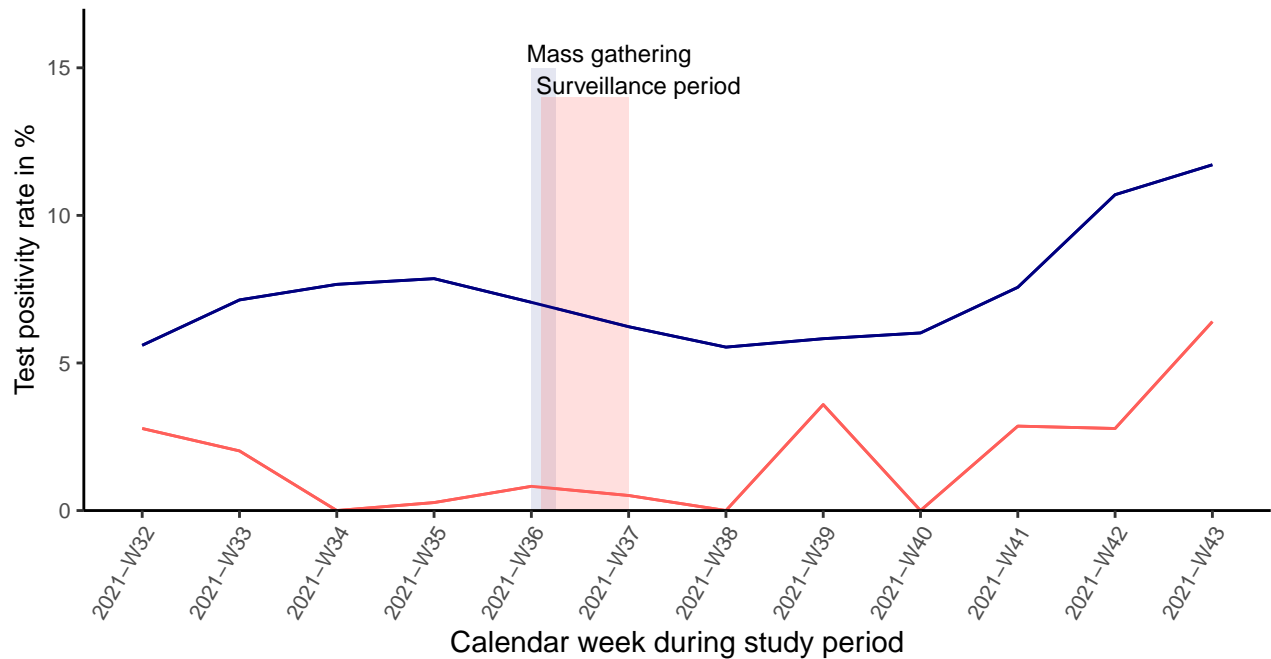

Supplement: Supplementary file 1 — Additional file 1: [file 12889_2022_14505_MOESM1_ESM.pdf]
